# Sociodemographic Correlates of Breast Cancer Screening Beliefs and Barriers Among Women in Kuwait: A Cross-Sectional Study Using the BCSBQ

**DOI:** 10.3390/healthcare14121711

**Published:** 2026-06-15

**Authors:** Fatima Al-Ghadban, Ahmad Salman, Ahmad Abbas

**Affiliations:** 1 Department of Public Health Practice, College of Public Health, Kuwait University, Sabah Al-Salem University City, Safat 13110, Kuwait; ahmad.salman@ku.edu.kw; 2 Nuclear Medicine Department, Farwaniya Hospital, Ministry of Health, Kuwait City 13110, Kuwait; abbas_ahmad63@hotmail.com

**Keywords:** breast cancer screening, health beliefs, perceived barriers, mammography, fatalism, sociodemographic factors, Kuwait, health education, BCSBQ

## Abstract

**Highlights:**

**What are the main findings?**

- The BCSBQ demonstrated good reliability (Cronbach’s α = 0.81) and a clear three-factor structure (positive attitudes, fatalistic beliefs, mammography-related barriers) in this Kuwaiti sample, supporting its psychometric validity for use in Arabic-speaking Gulf populations.
- Symptom-triggered misconceptions were endorsed by more than half of participants (57.2%), and lower education and non-employment were independently associated with higher mammography-related barriers and overall adverse belief scores.

**What are the implications of the main findings?**

- Health-promotion campaigns within the Kuwait National Mammography Screening Program should explicitly counter symptom-driven misconceptions and modesty-related barriers through culturally tailored, Arabic-language messaging, especially for women with lower educational attainment.
- Researchers and policymakers can use the validated BCSBQ to monitor screening beliefs over time and to design and evaluate equity-focused interventions to improve participation in Kuwait’s free-access mammography program.

**Abstract:**

**Background/Objectives**: Despite the availability of free national mammography services, participation in breast cancer screening remains suboptimal in Kuwait. This study aimed to assess breast cancer screening beliefs and barriers among women in Kuwait using the Breast Cancer Screening Beliefs Questionnaire (BCSBQ) and to examine their associations with sociodemographic characteristics. **Methods**: This study is a secondary analysis of a cross-sectional dataset collected among 458 women aged ≥20 years across all six governorates of Kuwait. Data were collected using a structured questionnaire including the BCSBQ, breast cancer knowledge measures, and a general help-seeking barrier scale. Internal consistency was assessed using Cronbach’s alpha, and exploratory factor analysis was performed to evaluate construct validity. Group differences were examined using *t*-tests and one-way analysis of variance with post hoc correction, while adjusted associations were assessed using general linear models and linear regression. **Results**: The BCSBQ demonstrated good internal consistency (Cronbach’s α = 0.808) and a clear three-factor structure (Kaiser–Meyer–Olkin = 0.790; Bartlett’s test *p* < 0.001), explaining 50.26% of the total variance. Nearly half of participants (48.7%) reported positive attitudes toward screening, while 16.4% exhibited firmer fatalistic beliefs and 18.8% reported elevated mammography-related barriers. A substantial proportion believed that screening is only necessary when symptoms are present (57.2%). Lower education and younger age were significantly associated with higher barriers and less favorable screening beliefs (*p* < 0.05). In regression analysis, higher mammography-related barriers were associated with greater general help-seeking barriers, whereas more positive attitudes were associated with lower barriers, explaining 11.5% of the variance (R^2^ = 0.115, *p* < 0.001). **Conclusions**: Breast cancer screening beliefs among women in Kuwait are shaped by sociodemographic factors, particularly education level and age, with persistent misconceptions and perceived barriers influencing screening perceptions. Targeted, culturally appropriate interventions are needed to address these gaps and promote participation in Kuwait’s free-access screening program.

## 1. Introduction

Breast cancer remains the most commonly diagnosed malignancy among women worldwide, having surpassed lung cancer as the leading cancer by incidence, with an estimated 2.3 million new cases recorded globally in 2020 [1]. Despite advances in treatment, it continues to represent a substantial cause of cancer-related mortality, with incidence projected to rise further in the coming decades [1,2]. In the Gulf Cooperation Council (GCC) region, breast cancer incidence has increased steadily over recent decades, with a trend toward diagnosis at younger ages compared to Western populations [2,3]. In Kuwait, breast cancer is the most frequently diagnosed malignancy among women, representing approximately 39.8% of all female cancer cases, highlighting the critical importance of effective early detection strategies [4]. 

Early detection through organized mammography screening has been consistently shown to reduce breast cancer mortality by enabling diagnosis at earlier, more treatable stages [5,6]. In recognition of this evidence, the Kuwait Ministry of Health, in collaboration with Memorial Sloan Kettering Cancer Center, launched the Kuwait National Mammography Screening Program (KNMSP) in 2014, offering free biennial digital mammography to asymptomatic women aged 40–69 years across Kuwait’s health regions [4,7]. Despite the elimination of financial barriers, a critical facilitator of access in many settings, participation has remained persistently low, with only 7.8% of the target population screened over the program’s first five years, far below the 70% coverage target [4]. This gap between availability and uptake suggests that non-financial factors play a key role in shaping screening behavior. 

A growing body of evidence indicates that women’s beliefs, attitudes, and perceptions play a critical role in shaping screening behavior [8,9]. Misconceptions about breast cancer risk, fatalistic beliefs, and reliance on symptom-driven healthcare-seeking have been consistently associated with lower screening participation [10,11]. Fatalistic beliefs, defined as the perception that cancer outcomes are predetermined and beyond individual control, are particularly relevant in Arab and Muslim-majority populations, where cultural and religious frameworks may shape health perceptions [11,12]. In addition, perceived barriers such as embarrassment, fear of diagnosis, concerns about breast exposure, and logistical challenges associated with mammography have been documented as significant deterrents to screening engagement in the GCC region and across the broader Arab world [12,13]. 

The Health Belief Model (HBM) provides a well-established theoretical framework for understanding these dynamics, positing that health behavior is shaped by perceived susceptibility, perceived severity, perceived benefits, and perceived barriers [8,9,14]. Within this framework, fatalistic beliefs may reduce perceived susceptibility and perceived benefit, while modesty-related concerns increase perceived barriers, thereby discouraging screening participation [8,9,15]. Studies conducted in Qatar, Saudi Arabia, and among Arabic-speaking women in Australia have demonstrated that these belief-based barriers persist even among women with adequate knowledge of breast cancer, suggesting that awareness alone may be insufficient without also addressing underlying belief systems [12,13,16]. 

Sociodemographic characteristics, including age, education level, employment status, and personal or family history of breast cancer, have also been shown to influence breast cancer screening beliefs and practices [17,18]. Women with lower educational attainment often exhibit more misconceptions, greater perceived barriers, and less favorable attitudes toward preventive screening [18,19]. Similarly, younger women may underestimate their risk or perceive screening as unnecessary in the absence of symptoms, contributing to delayed engagement with screening programs [20]. This pattern has been observed among women under 40 years of age, that is, below the recommended screening initiation age threshold defined in major guidelines, including the American Cancer Society guidelines for women aged 40 years and older [20]. Understanding how these sociodemographic factors interact with belief systems within Kuwait’s specific cultural and healthcare context is therefore essential for designing targeted and resource-efficient interventions. 

To systematically assess multidimensional screening beliefs, the Breast Cancer Screening Beliefs Questionnaire (BCSBQ) was developed by Kwok and colleagues and has since been applied and validated across diverse cultural contexts, including Chinese-Australian, Arabic-Australian, African-Australian, Korean, Iranian, and Greek populations [16,21]. The BCSBQ captures three core domains, namely positive attitudes toward screening, fatalistic beliefs, and perceived barriers to mammography, providing a comprehensive framework for examining screening-related beliefs. Its Arabic version has demonstrated good psychometric properties, with Cronbach’s alpha values ranging from 0.81 to 0.93 across subscales [16]. However, its application among women residing in Kuwait and the broader GCC region remains limited, and the sociodemographic patterning of BCSBQ-measured beliefs in this population has not been systematically examined. Addressing this gap is essential to inform culturally appropriate health promotion strategies within Kuwait’s free-access screening system. 

Therefore, through secondary analysis of an existing national cross-sectional dataset, this study aimed to assess breast cancer screening beliefs and barriers among women in Kuwait using the BCSBQ, to evaluate its internal consistency and factor structure, and to examine sociodemographic correlates of these beliefs and their association with general help-seeking barriers. Building on the conceptual framework outlined above, we hypothesized that (i) symptom-triggered misconceptions and perceived mammography barriers would be the most prevalent adverse belief patterns; (ii) lower educational attainment would be associated with less favorable belief profiles and higher perceived barriers; and (iii) mammography-specific barriers would be independently associated with greater general help-seeking barriers after adjustment for sociodemographic covariates.

## 2. Materials and Methods

### 2.1. Study Design and Data Source

This study represents a secondary analysis of a previously conducted cross-sectional survey designed and implemented by the authors. The original parent study (Ministry of Health, Kuwait, Research No. 2024/2608) was designed to evaluate breast cancer screening knowledge, attitudes, beliefs, and practices (KABP) among women in Kuwait, with primary outcomes including awareness of the Kuwait National Mammography Screening Program (KNMSP), breast cancer warning-sign knowledge, and screening behaviors (breast self-examination, clinical breast examination, and mammography). The original survey was conducted between January and April 2024 among women aged 20 years or older residing across all six governorates of Kuwait, using a structured, self-administered questionnaire distributed via online platforms and community networks. The dataset included comprehensive information on sociodemographic characteristics, breast cancer awareness, screening behaviors, perceived barriers, and health-related beliefs. The present secondary analysis focuses specifically on breast cancer screening beliefs and perceived barriers, with additional emphasis on evaluating the psychometric properties of the belief constructs and examining their associations with sociodemographic factors and general help-seeking barriers.

### 2.2. Study Population

The analytical sample consisted of 458 women aged 20 years or older who met the eligibility criteria and completed the original survey. Inclusion criteria were female sex, age ≥ 20 years, and current residence in Kuwait. Only participants with complete data on key variables relevant to the present analysis were included. Participants were recruited using a convenience sampling strategy. The structured, self-administered questionnaire was disseminated in parallel Arabic- and English-language versions through multiple channels to maximize reach across all six governorates, including social-media platforms (WhatsApp, Instagram, Twitter/X), women’s health advocacy networks, and institutional mailing lists. Quick-Response (QR) codes linking directly to the survey were physically distributed in hospitals, primary health centers, and university campuses to reach women with varying levels of digital access. Community organizations and public health networks were also engaged to support recruitment across diverse age groups, educational levels, and governorates. 

Of 516 women who accessed the questionnaire, 58 were excluded for failing to meet eligibility criteria or for providing incomplete responses (≥20% missing items), yielding 458 complete cases, corresponding to an 88.7% completion rate among those who began the survey. A formal response rate cannot be calculated for this online convenience sample, as the total number of individuals exposed to the survey link cannot be precisely determined. No formal a priori sample size calculation was performed for the parent KABP study, which was descriptive and exploratory in design. The questionnaire was available in parallel Arabic- and English-language versions, with 424 and 34 participants completing the Arabic and English versions, respectively. The instrument underwent content validation by three public health and oncology experts and was pilot-tested on 30 participants prior to field deployment to confirm clarity, cultural appropriateness, and linguistic accuracy.

### 2.3. Measures

#### 2.3.1. Sociodemographic Characteristics

Variables included age, nationality, governorate of residence, education level, employment status, marital status, number of children, and personal and family history of breast cancer.

#### 2.3.2. Breast Cancer Screening Beliefs

Breast cancer screening beliefs were assessed using the Breast Cancer Screening Beliefs Questionnaire (BCSBQ), a validated instrument originally developed by Kwok and colleagues [21] and subsequently validated in Arabic-speaking populations [16]. The BCSBQ comprises 13 items across three subscales: (1) positive attitudes toward screening (4 items), reflecting engagement with preventive health check-ups in the absence of symptoms; (2) fatalistic beliefs about breast cancer (4 items), capturing cultural and illness-related perceptions that cancer is predetermined or incurable; and (3) perceived barriers to mammography (5 items), covering psychological and practical obstacles to screening participation. 

Within the positive attitudes subscale, items BCSB1 (“I feel well, so I do not need screening”) and BCSB4 (“I feel healthy, so I do not need screening”) tap related but conceptually distinct facets of the same misconception. BCSB1 anchors the belief in the current symptom-free physical state, whereas BCSB4 reflects a broader general health identity. Both items contribute to the symptom-triggered underestimation of the need for preventive screening. Items were rated on a five-point Likert scale (1 = strongly disagree to 5 = strongly agree). Reverse coding was applied where appropriate following established methodology [16,21,22], and subscale scores were calculated as mean values, with higher scores indicating stronger endorsement of the underlying construct. A composite adverse belief score was also computed by summing dichotomized item responses (agree vs. disagree/neutral) across all 13 items. This approach was used to facilitate the interpretation of unfavorable belief profiles at the population level.

#### 2.3.3. Breast Cancer Knowledge

Breast cancer knowledge was assessed using adapted modules from the Cancer Awareness Measure (CAM) [23], a validated tool for assessing cancer awareness in the general population. The questionnaire included nine items assessing awareness of warning signs and eleven items assessing knowledge of risk factors. Scoring followed established CAM procedures, with higher scores indicating greater awareness.

#### 2.3.4. General Help-Seeking Barriers

Perceived barriers to seeking medical care were assessed using CAM-based items [23], capturing emotional, practical, and communication-related barriers to seeking healthcare. A composite barrier score was calculated, with higher values indicating greater perceived difficulty in seeking medical care (possible range: 0–20).

### 2.4. Variable Construction

Derived variables were created to facilitate statistical analysis. Age was categorized as <40 years and ≥40 years. Education level was dichotomized as less than a bachelor’s degree versus bachelor’s degree or higher. Employment was categorized as employed versus not employed. BCSBQ subscale scores were treated as continuous variables in all primary analyses. Additional binary categorizations of individual BCSBQ items (agree vs. disagree/neutral) were generated for item-level and chi-square analyses.

### 2.5. Statistical Analysis

Data were analyzed using IBM SPSS Statistics, Version 31 (IBM Corp., Armonk, NY, USA). Statistical significance was set at *p* < 0.05 (two-tailed). All analyses were conducted using complete-case data. No imputation was performed for missing data. The distribution of BCSBQ subscale scores was assessed for normality using the Shapiro–Wilk and Kolmogorov–Smirnov (Lilliefors) tests and visual inspection of Q–Q plots. Consistent with the ordinal nature of Likert-derived scores, the subscale distributions deviated from normality (Shapiro–Wilk all *p* < 0.001). However, given the sample size (*N* = 458), parametric tests (independent-samples *t*-tests, one-way ANOVA, linear regression) were employed in accordance with the central limit theorem, which supports the robustness of parametric inference at this sample size. To verify the robustness of group comparisons, Mann–Whitney U sensitivity analyses were conducted for the principal between-group findings reported in Section 3.5; these yielded substantively identical conclusions. Effect sizes were reported alongside *p*-values: Cohen’s d for between-group comparisons and partial η^2^ for general linear model effects.

#### 2.5.1. Descriptive Analysis

Descriptive statistics were used to summarize participant characteristics and study variables. Continuous variables were reported as means and standard deviations, and categorical variables as frequencies and percentages. Item-level endorsement frequencies were reported for all 13 BCSBQ items.

#### 2.5.2. Reliability and Construct Validity

Internal consistency of the BCSBQ and its subscales was assessed using Cronbach’s alpha, with values of α ≥ 0.70 considered acceptable. Exploratory factor analysis (EFA) was conducted using principal axis factoring with oblique (Direct Oblimin) rotation, following established methodological approaches for scale validation [21]. Sampling adequacy was assessed using the Kaiser–Meyer–Olkin (KMO) measure, and Bartlett’s test of sphericity was used to confirm the factorability of the correlation matrix. Factors were retained based on eigenvalues greater than 1.0 and interpretability of the factor solution. Pearson correlation coefficients were computed to examine associations between BCSBQ subscales and the composite adverse belief score.

#### 2.5.3. Bivariate Analysis

Independent-samples *t*-tests were used to examine differences in BCSBQ subscale scores across binary sociodemographic groups. One-way analysis of variance (ANOVA) with Bonferroni post hoc correction was applied for multi-category comparisons. Chi-square tests were used to examine item-level differences in dichotomized BCSBQ responses across sociodemographic subgroups as supplementary analyses.

#### 2.5.4. Multivariable Analysis

General linear models (GLMs) were used to examine adjusted associations between sociodemographic factors and BCSBQ subscale scores, with simultaneous adjustment for age, nationality, education level, and marital status. Multiple linear regression analysis was conducted to identify factors associated with general help-seeking barriers (total CAM barrier score), with BCSBQ subscale scores and breast cancer knowledge scores entered as predictors. The coefficient of determination (R^2^) is reported as the proportion of variance explained. Model assumptions, including normality of residuals and homoscedasticity, were assessed and met.

### 2.6. Ethical Considerations

Ethical approval for the original study was obtained from the Ministry of Health, Kuwait (Research No. 2024/2608, approved on 29 August 2024). The present study constitutes a secondary analysis of anonymized data. All procedures complied with ethical standards in accordance with the Declaration of Helsinki, and informed consent had been obtained from all participants in the original study.

## 3. Results

### 3.1. Participant Characteristics

A total of 458 women were included in the analysis. Participants were recruited from all six governorates of Kuwait, with the largest proportions from Hawally (28.4%), Al Asima (20.5%), and Mubarak Al Kabeer (19.9%), ensuring broad geographic representation. As only categorical age was captured in the parent dataset, a continuous mean age cannot be reported. Participants were approximately evenly distributed across the four age categories (20–29 years: 27.9%; 30–39 years: 28.4%; 40–49 years: 28.6%; ≥50 years: 15.1%), with 43.7% of respondents aged 40 years or older, placing them within the KNMSP eligibility range. The majority of respondents completed the Arabic version of the questionnaire (*n* = 424, 92.6%), while 34 (7.4%) completed the English version. Detailed sociodemographic characteristics are presented in Table 1.

### 3.2. Distribution of Breast Cancer Screening Beliefs

Overall, participants demonstrated moderate levels across all belief domains. The positive attitudes subscale had a mean of 2.89 (SD = 1.20), the fatalistic beliefs subscale a mean of 1.91 (SD = 0.95), and the mammography-related barriers subscale a mean of 2.05 (SD = 0.96). The overall adverse belief score had a mean of 30.31 (SD = 9.63). Based on midpoint classification, 48.7% of participants demonstrated favorable attitudes toward screening, 16.4% exhibited elevated fatalistic beliefs, and 18.8% reported elevated mammography-related barriers.

At the item level, the most commonly endorsed adverse belief was that screening is only necessary when symptoms are present (BCSB3; 57.2%). Additionally, 39.1% believed that feeling well removes the need for screening (BCSB1), and 29.5% believed that following a healthy lifestyle removes the need for screening (BCSB2). Among barrier-related items, 27.9% endorsed embarrassment as a concern (BCSB13), and 20.7% reported discomfort with breast exposure during mammography (BCSB12). Item-level distributions are presented in Table 2.

### 3.3. Reliability and Construct Validity of the BCSBQ

The BCSBQ demonstrated good internal consistency (Table 3). The full 13-item scale yielded Cronbach’s alpha of 0.808. Subscale alphas were 0.839 for positive attitudes, 0.776 for fatalistic beliefs, and 0.760 for mammography-related barriers, all meeting the threshold of α ≥ 0.70.

Exploratory factor analysis supported a three-factor structure consistent with the instrument’s theoretical domains. The KMO value was 0.790, and Bartlett’s test of sphericity was statistically significant (*p* < 0.001), confirming sampling adequacy and factorability. The three factors jointly explained 50.26% of total variance, corresponding to fatalistic beliefs, positive attitudes toward screening, and mammography-specific barriers. Primary factor loadings ranged from 0.35 to 0.91, with limited cross-loading, supporting the construct validity of the BCSBQ in this Kuwaiti sample.

### 3.4. Correlations Between Belief Domains

Pearson correlation analysis revealed significant associations between all BCSBQ belief domains (Table 4). Positive attitudes were negatively correlated with fatalistic beliefs (r = −0.169, *p* < 0.01; small effect) and with mammography barriers (r = −0.250, *p* < 0.01; small effect). Fatalism was positively correlated with barriers (r = 0.399, *p* < 0.001; moderate effect). The overall adverse belief score was negatively correlated with positive attitudes (r = −0.690, *p* < 0.001), positively correlated with fatalism (r = 0.678, *p* < 0.001), and most strongly correlated with mammography barriers (r = 0.779, *p* < 0.001). Effect-size labels follow Cohen’s conventions for correlation coefficients (small: |r| ≈ 0.10–0.29; moderate: 0.30–0.49; large: ≥0.50). Although the intercorrelations among subscales were statistically significant, several were small in magnitude and should be interpreted in light of the large sample size, which enhances statistical power to detect even modest associations.

### 3.5. Group Differences in Screening Beliefs

Independent-samples *t*-tests revealed significant differences in selected BCSBQ subscale scores across sociodemographic groups (Table 5). Women aged 40 years or older reported significantly more favorable attitudes toward screening compared to younger women (mean 3.06 vs. 2.76; *p* = 0.008; Cohen’s d = 0.25, small), although the effect size was small. Participants with less than a bachelor’s degree reported significantly higher mammography-related barriers (mean 2.27 vs. 1.93; *p* < 0.001; d = 0.36, small) and overall adverse belief scores (mean 31.57 vs. 29.66; *p* = 0.045; d = 0.20, small) compared to those with a bachelor’s degree or higher. Employed women reported significantly lower perceived barriers compared to non-employed women (mean 1.93 vs. 2.25; *p* < 0.001; d = 0.34, small). No statistically significant differences were observed by nationality or family history of breast cancer (all *p* > 0.05; all |d| < 0.20, negligible). 

Regarding family history specifically, women reporting a positive family history (*n* = 14) showed slightly higher mean adverse belief scores compared to women reporting no family history (*n* = 444; mean 32.29 vs. 30.24; *p* = 0.435; d = 0.21); however, the small size of the positive-history subgroup substantially limits the statistical power and precision of this comparison. With the exception of the education-related barrier effect (d = 0.36), all between-group effect sizes were negligible to small (d < 0.40), indicating that statistically significant differences corresponded to modest practical magnitudes. 

Mann–Whitney U sensitivity analyses confirmed the *t*-test conclusions for all four primary findings (education–barriers: *p* = 0.002; education–adverse: *p* = 0.037; age–attitudes: *p* = 0.016; employment–barriers: *p* < 0.001), supporting the robustness of these results despite the non-normal distribution of subscale scores. Additional one-way ANOVA analyses indicated significant differences across education levels for mammography barriers and adverse beliefs, with Bonferroni post hoc comparisons suggesting less favorable belief profiles among lower-educational-attainment groups.

### 3.6. Multivariable Analysis (General Linear Model)

In the adjusted general linear model, education level remained a significant independent predictor of mammography-related barriers and overall adverse beliefs, with small effect sizes (partial η^2^ ≈ 0.02). Age showed a modest association with positive attitudes toward screening (partial η^2^ ≈ 0.005), indicating a small effect. Nationality and marital status were not significantly associated with any belief subscale after adjustment. Overall, the sociodemographic variables examined explained only a limited proportion of the variance in screening beliefs, suggesting that additional psychosocial and contextual factors, beyond demographic characteristics, contribute meaningfully to belief formation.

### 3.7. Factors Associated with General Help-Seeking Barriers

Multiple linear regression analysis was conducted to identify factors associated with general help-seeking barriers. The overall model was statistically significant (F(9, 448) = 6.48, *p* < 0.001) and explained 11.5% of the variance in general help-seeking barriers (R^2^ = 0.115). For clarity, BCWS refers to breast cancer warning sign knowledge score, and CDCA refers to cancer delay and care avoidance.

Higher mammography-related barriers were the strongest predictor of increased general help-seeking barriers (β = 0.223, *p* < 0.001). More favorable attitudes toward screening were significantly associated with lower general barriers (β = −0.132, *p* = 0.005). Higher breast cancer knowledge scores were also positively associated with greater general barriers (β = 0.115, *p* = 0.021). Fatalistic beliefs and CDCA scores were not significantly associated with general help-seeking barriers. Age of 40 years or older showed a borderline negative association (*p* = 0.056), suggesting a potential trend toward lower perceived barriers among older women. Full regression coefficients are presented in Table 6.

### 3.8. Item-Level Sociodemographic Differences

Chi-square analyses revealed significant differences in selected BCSBQ item responses across sociodemographic subgroups. Education-related differences were observed for mammography barrier items, including transportation difficulties and language barriers, with women with lower educational attainment more likely to endorse these concerns. Age-related differences were observed for modesty-related barrier items, with younger women more likely to report discomfort with breast exposure during mammography (BCSB12; *p* = 0.015). Nationality was not significantly associated with most individual belief items. Detailed item-level results are presented in Supplementary Table S1.

## 4. Discussion

### 4.1. Principal Findings

This study provides a comprehensive assessment of breast cancer screening beliefs among women in Kuwait using the Breast Cancer Screening Beliefs Questionnaire (BCSBQ), a validated multidimensional instrument, and examines its psychometric properties, sociodemographic correlates, and associations with general help-seeking barriers. As a secondary analysis of a national cross-sectional dataset covering all six governorates of Kuwait, the findings offer important insights into the belief structures underlying persistently low participation in the Kuwait National Mammography Screening Program (KNMSP), despite its availability at no cost [4].

Overall, the results reveal a moderate but concerning belief profile, characterized by relatively favorable attitudes toward screening alongside persistent misconceptions and non-negligible perceived barriers. Nearly half of the participants demonstrated favorable attitudes; however, a substantial proportion endorsed symptom-driven beliefs, with more than half indicating that screening is only necessary when health problems arise. This highlights a critical disconnect between awareness and appropriate preventive behavior, consistent with prior evidence that awareness alone does not reliably translate into screening uptake [19,20]. Fatalistic beliefs were present but relatively low in prevalence compared to other misconceptions and perceived barriers, suggesting that structural and cognitive misunderstandings about the purpose of screening, rather than fatalism alone, may be the primary driver of avoidance in this population. Nonetheless, modesty-related concerns, including embarrassment and discomfort with breast exposure, remained salient, reinforcing the role of culturally embedded factors in shaping screening behavior [11,13].

### 4.2. Comparison with Existing Literature

The predominance of symptom-triggered beliefs observed in this study is consistent with findings from comparable GCC and Arab populations, where preventive screening is widely underutilized in the absence of symptoms, reflecting a broader reliance on reactive rather than preventive healthcare-seeking behavior [11,13]. Within the Health Belief Model (HBM) framework, this pattern reflects low perceived susceptibility, specifically the belief that risk only materializes through symptoms, combined with limited perceived benefit of routine asymptomatic screening [8,9].

The relatively low prevalence of strong fatalistic beliefs contrasts with earlier literature that identified cancer fatalism as a primary barrier among Arab women [24]. This may reflect a gradual shift toward improved cancer literacy and changing perceptions over time, although fatalistic beliefs remain interlinked with other barriers, particularly fear and perceived lack of personal control [15]. Importantly, the significant positive correlation between fatalistic beliefs and mammography barriers (r = 0.399, *p* < 0.001) confirms that these domains co-occur and may jointly impede screening engagement, consistent with HBM predictions that perceived barriers represent the most powerful single determinant of preventive health behavior [8,9].

The psychometric findings of this study provide important support for the cross-cultural applicability of the BCSBQ in an Arabic-speaking Gulf population. The confirmed three-factor structure, KMO of 0.790, total variance explained of 50.26%, and Cronbach’s alpha values ranging from 0.760 to 0.839 are consistent with values reported in the Arabic-Australian validation study [16] and with psychometric evaluations of the BCSBQ across other cultural contexts. To our knowledge, this represents the first application of the BCSBQ among women residing in Kuwait and the broader GCC region, contributing a validated measurement resource for future belief-focused research in this setting.

The observed sociodemographic gradients, particularly the influence of education on mammography barriers and adverse beliefs, are consistent with global evidence demonstrating that lower educational attainment is associated with poorer cancer awareness, higher perceived barriers, and lower screening participation [18,19]. Notably, nationality was not significantly associated with any BCSBQ subscale, suggesting that shared healthcare structures and cultural context in Kuwait may attenuate differences between Kuwaiti and non-Kuwaiti residents in this domain.

### 4.3. Interpretation and Theoretical Implications

From a theoretical standpoint, the findings reinforce the relevance of the HBM in explaining breast cancer screening behavior, while also highlighting its limitations when applied in isolation [8,9]. The regression analysis indicates that mammography-specific barriers are the strongest independent predictor of general help-seeking barriers (β = 0.223, *p* < 0.001), while more positive screening attitudes are associated with lower general barriers (β = −0.132, *p* = 0.005). This extends prior evidence suggesting that perceived barriers function not only as screening-specific deterrents but as part of broader tendencies to delay or avoid medical care more generally [12].

The coexistence of moderately favorable attitudes with widespread symptom-triggered misconceptions supports the well-established principle that knowledge and awareness alone are insufficient to drive behavioral change [19,20]. Women may recognize the value of screening yet delay or avoid participation due to entrenched beliefs or emotional concerns. This highlights the need for behaviorally informed interventions that move beyond awareness campaigns to directly target decision-making processes and the emotional determinants of participation.

The clustering of fatalistic beliefs and perceived barriers as intercorrelated constructs, rather than independent deterrents, suggests that integrated interventions addressing both cognitive and emotional dimensions simultaneously may be more effective than single-domain approaches. The unexpected positive association between breast cancer knowledge and general help-seeking barriers warrants cautious interpretation; it may reflect a heightened awareness effect, whereby women with greater cancer knowledge are more attuned to the perceived complexities of navigating healthcare systems, or it may indicate residual confounding by unmeasured psychosocial factors. 

The magnitude of associations observed in this analysis warrants careful interpretation. With a sample of 458 women, parametric tests have substantial statistical power to detect even small effects, and the majority of significant between-group differences in Table 5 correspond to Cohen’s d values below 0.40, indicating that statistical significance reflects relatively modest practical differences in belief profiles. Similarly, the regression model explained 11.5% of the variance in general help-seeking barriers, suggesting that screening beliefs are one of many contributors to broader healthcare avoidance patterns. The findings should therefore be interpreted as identifying targets for further investigation and intervention design, rather than as quantitatively decisive determinants of screening behavior.

### 4.4. Public Health and Policy Implications

The findings have several direct implications for breast cancer screening strategies in Kuwait and similar free-access, low-uptake settings.

First, the high prevalence of symptom-driven beliefs highlights the need to reframe public health messaging to explicitly communicate the importance and rationale of asymptomatic, routine screening [5,6]. Campaigns should directly counter the misconception that feeling well removes the need for mammography.

Second, the persistence of modesty-related and emotional barriers indicates that culturally sensitive service delivery models are essential, including access to female providers, women-only screening sessions, and privacy assurances [11,13].

Third, the strong association between screening barriers and general help-seeking barriers suggests that interventions should adopt a system-wide perspective to address broader challenges in communication quality, patient trust, and healthcare access [12].

Fourth, education-related disparities in belief profiles suggest the need for targeted outreach to women with lower educational attainment, using simplified messaging, Arabic-language communication, and community-based engagement strategies [18,19].

Fifth, given the KNMSP’s participation rate of approximately 7.8% of the eligible population [4], implementing evidence-based, belief-targeted behavioral strategies represents an urgent and actionable public health priority.

### 4.5. Strengths and Limitations

This study has several notable strengths. It represents one of the first comprehensive applications of the BCSBQ in a Kuwaiti population, integrating psychometric characterization with analysis of belief patterns and help-seeking barriers [21]. The geographically diverse sample spanning all six governorates enhances national representativeness, and the use of a validated multidimensional instrument supports comparability with international literature.

Several limitations should be acknowledged. The cross-sectional design precludes causal inference. First and most importantly, the online convenience sampling strategy introduced systematic selection bias: more than half of the sample (56.3%) was under 40 years of age—below the KNMSP screening-eligible age threshold—and approximately two-thirds (66.2%) held a bachelor’s degree or higher. This sociodemographic profile diverges substantially from the demographic composition of the KNMSP target population (women aged 40–69 years, with a broader educational distribution). The findings should be interpreted as representative of the study sample rather than generalizable to all Kuwaiti women or the KNMSP-eligible population. Inferences drawn from this study should be considered hypothesis-generating for the broader target population and confirmed in samples that more closely reflect the age and educational distributions of women within the KNMSP age range. The online dissemination strategy may introduce selection bias, potentially over-representing women with higher digital literacy and education, the very groups with more favorable belief profiles, thereby limiting generalizability to women with the lowest educational attainment. Second, although the sample size of 458 participants is adequate for the primary analyses, it limits the statistical power and precision of certain subgroup estimates, most notably the non-Kuwaiti subgroup (*n* = 57) and the family history-positive subgroup (*n* = 14), and findings from these smaller subgroups should be interpreted with caution. Third, while several between-group differences were statistically significant, the corresponding effect sizes were small (Cohen’s d ranging from 0.20 to 0.36), and the regression model explained only 11.5% of the variance in general help-seeking barriers. These modest effect sizes underscore that screening beliefs account for only a portion of the variation in healthcare-seeking patterns, and that practical magnitude—not statistical significance alone—should guide interpretation of the findings. Fourth, the present analysis examines belief profiles but does not link these to objective mammography uptake; consequently, the relationship between the belief patterns reported here and the persistently low KNMSP participation rate of 7.8% remains inferential rather than empirically demonstrated. Self-reported measures are also subject to recall and social desirability biases. Although exploratory factor analysis confirmed the three-factor structure of the BCSBQ, confirmatory factor analysis in an independent sample would further strengthen the psychometric evidence base. Finally, the absence of linkage to objective screening records from the KNMSP limits direct assessment of how belief profiles translate into actual screening behavior.

### 4.6. Future Research Directions

Future studies should employ longitudinal designs to examine causal pathways between belief profiles and screening participation, ideally integrating objective screening data from national program registries. Prospective cohorts that link BCSBQ-measured belief profiles to KNMSP electronic screening records would directly test the predictive value of belief-based variables for actual mammography uptake, a question that the present cross-sectional dataset cannot resolve. Targeted sampling of women aged 40–69 years (the KNMSP-eligible age band) and stratified recruitment by educational level would generate population-representative estimates more suitable for programmatic planning. Intervention studies are needed to evaluate which belief-modifying strategies, including narrative-based health communication, community health worker programs, and motivational interviewing, yield measurable improvements in mammography uptake. Qualitative research could provide deeper insight into the cultural and social dynamics shaping women’s perceptions of screening, particularly among subgroups with the least favorable belief profiles. Confirmatory factor analysis of the BCSBQ in future Kuwaiti samples would consolidate the psychometric evidence established by this study.

## 5. Conclusions

This study provides one of the first comprehensive evaluations of breast cancer screening beliefs among women in Kuwait using a validated, multidimensional framework. Despite the availability of a free national mammography screening program, participation remains low, and the present findings, interpreted within the constraints of an online convenience sample, highlight the critical role of belief systems in shaping screening behavior.

While general attitudes toward screening were moderately favorable, substantial proportions of women endorsed symptom-driven beliefs and reported persistent emotional and cultural barriers to mammography. In contrast, fatalistic beliefs were less prominent, suggesting that misconceptions about the purpose of screening and perceived practical and emotional barriers may play a more influential role than fatalism in this context. The BCSBQ demonstrated good reliability and a confirmed three-factor structure, supporting its validity for use in Arabic-speaking Gulf populations. The strong association between mammography-specific barriers and general help-seeking barriers further indicates that screening behavior is embedded within broader patterns of healthcare utilization.

Sociodemographic disparities, particularly by education level, observed in this sample highlight the importance of targeted, equity-focused interventions, while recognizing that these patterns require confirmation in samples representative of the KNMSP-eligible population. The findings also reinforce that awareness alone is insufficient to drive screening uptake, emphasizing the need for behaviorally informed strategies that address underlying beliefs, emotional concerns, and decision-making processes.

From a public health perspective, improving breast cancer screening uptake in Kuwait will require a shift from awareness-based approaches toward culturally sensitive, belief-targeted interventions, supported by system-level enhancements that facilitate access and patient engagement. Addressing symptom-driven misconceptions, reducing perceived barriers, and strengthening trust in screening services represent critical and actionable steps to enhance early detection and reduce the burden of breast cancer among women in Kuwait.

## Figures and Tables

**Table 1:** Sociodemographic Characteristics of Participants (*N* = 458).

Variable	Category	*n* (%)
Age group	20–29 years	128 (27.9)
	30–39 years	130 (28.4)
	40–49 years	131 (28.6)
	≥50 years	69 (15.1)
Age (binary)	<40 years	258 (56.3)
	≥40 years	200 (43.7)
Nationality	Kuwaiti	401 (87.6)
	Non-Kuwaiti	57 (12.4)
Governorate	Al Asima	94 (20.5)
	Hawally	130 (28.4)
	Al Farwaniya	84 (18.3)
	Al Ahmadi	33 (7.2)
	Mubarak Al Kabeer	91 (19.9)
	Al Jahra	26 (5.7)
Education level	Less than high school	10 (2.2)
	High school	65 (14.2)
	Associate’s degree	80 (17.5)
	Bachelor’s degree	235 (51.3)
	Master’s/PhD	68 (14.8)
Education (binary)	Less than bachelor’s	155 (33.8)
	Bachelor’s or higher	303 (66.2)
Employment status	Full-time employee	269 (58.7)
	Part-time employee	22 (4.8)
	Retired/not employed	167 (36.5)
Marital status	Single	145 (31.7)
	Married	276 (60.3)
	Divorced or widowed	37 (8.1)
Personal history of breast cancer	No	430 (93.9)
	Yes	28 (6.1)
Family history of breast cancer	No	417 (91.0)
	Yes	14 (3.1)
	Don’t know	27 (5.9)

Note. Age was collected only as a categorical variable in the parent dataset; continuous age values are therefore unavailable. The four age categories shown above (20–29, 30–39, 40–49, and ≥50 years) approximate a symmetric distribution across the adult-female age range represented in the sample, with the median falling within the 30–39 years category.

**Table 2:** Item-Level Distribution of BCSBQ Beliefs (*N* = 458).

Item	Statement (Original Wording)	Mean	Endorsed (%)
Items contributing to the Positive Attitudes factor (original wording before reverse scoring)			
BCSB1	I feel well, so I do not need screening	3.05	39.1
BCSB2	I follow a healthy lifestyle, so I do not need screening	2.67	29.5
BCSB3	I only go for screening when I have health problems	3.52	57.2
BCSB4	I feel healthy, so I do not need screening	3.19	44.8
Fatalistic Beliefs			
BCSB5	Breast cancer is a life-or-death sentence	1.83	10.0
BCSB6	Breast cancer cannot be cured	1.77	9.2
BCSB7	There is little I can do to reduce my chances of dying from breast cancer	1.74	9.2
BCSB8	There is nothing I can do to change my fate	2.31	22.1
Mammography-Related Barriers			
BCSB9	A mammogram will hurt my breasts	2.36	21.6
BCSB10	It is difficult to arrange transportation for a mammogram	1.65	9.6
BCSB11	I cannot speak English	1.54	8.5
BCSB12	I need to expose my breasts for a mammogram	2.17	20.7
BCSB13	Having a mammogram is embarrassing	2.50	27.9

Note: Endorsed (%) reflects endorsement of “agree” or “strongly agree” on the original scale. Items BCSB1–BCSB4 were reverse-scored prior to subscale calculation.

**Table 3:** Internal Consistency of the BCSBQ (*N* = 458).

Scale/Subscale	Number of Items	Cronbach’s α
Full BCSBQ scale	13	0.808
Positive attitudes toward screening	4	0.839
Fatalistic beliefs	4	0.776
Mammography-related barriers	5	0.760

Note: EFA summary—KMO = 0.790; Bartlett’s test *p* < 0.001; total variance explained = 50.26%; three-factor solution with primary loadings ranging from 0.35 to 0.91.

**Table 4:** Pearson Correlations Between BCSBQ Belief Domains (*N* = 458).

Variable	1	2	3	4
1. Positive attitudes	1.000			
2. Fatalistic beliefs	−0.169 **	1.000		
3. Mammography barriers	−0.250 **	0.399 ***	1.000	
4. Overall adverse belief score	−0.690 ***	0.678 ***	0.779 ***	1.000

** *p* < 0.01; *** *p* < 0.001.

**Table 5:** Group Differences in BCSBQ Subscale Scores by Sociodemographic Characteristics (*N* = 458).

Variable	Group	*n*	Positive Attitudes Mean (SD)	*p*	Fatalism Mean (SD)	*p*	Barriers Mean (SD)	*p*	Adverse Score Mean (SD)	*p*
Age	<40 years	258	2.76 (1.12)	0.008	1.92 (0.94)	0.908	2.09 (0.94)	0.250	31.08 (8.90)	0.056
	≥40 years	200	3.06 (1.29)		1.91 (0.97)		1.99 (0.98)		29.31 (10.44)	
Nationality	Kuwaiti	401	2.87 (1.22)	0.341	1.92 (0.97)	0.825	2.03 (0.96)	0.360	30.32 (9.78)	0.913
	Non-Kuwaiti	57	3.04 (1.11)		1.89 (0.83)		2.15 (0.91)		30.18 (8.64)	
Education	<Bachelor’s	155	2.93 (1.17)	0.604	2.00 (0.98)	0.183	2.27 (1.06)	<0.001	31.57 (9.66)	0.045
	≥Bachelor’s	303	2.87 (1.22)		1.87 (0.94)		1.93 (0.88)		29.66 (9.57)	
Employment	Not employed	167	2.97 (1.18)	0.319	1.89 (0.91)	0.716	2.25 (1.00)	<0.001	30.92 (9.92)	0.300
	Employed	291	2.85 (1.22)		1.92 (0.98)		1.93 (0.91)		29.95 (9.46)	

*p*-values reflect independent-samples *t*-tests. Cohen’s d effect sizes for the significant comparisons: age–positive attitudes d = 0.25 (small); education–mammography barriers d = 0.36 (small); education–adverse score d = 0.20 (small); employment–mammography barriers d = 0.34 (small); all non-significant comparisons d < 0.20 (negligible). Cohen’s conventions: d < 0.20 negligible; 0.20–0.49 small; 0.50–0.79 medium; ≥0.80 large. Family history of breast cancer was assessed in a separate analysis (No, *n* = 444; Yes, *n* = 14): no statistically significant differences were observed across any BCSBQ subscale (positive attitudes *p* = 0.573; fatalism *p* = 0.836; mammography barriers *p* = 0.402; adverse score *p* = 0.435); the small “Yes” subgroup limits the precision of these estimates. Mann–Whitney U sensitivity analyses confirmed all four significant *t*-test findings (see Section 3.5).

**Table 6:** Linear Regression Analysis: Factors Associated with General Help-Seeking Barriers (*N* = 458).

Predictor	B	SE	β	*p*-Value
Positive attitudes toward screening	−0.304	0.107	−0.132	0.005
Fatalistic beliefs	0.095	0.142	0.033	0.505
Mammography-related barriers	0.645	0.145	0.223	<0.001
Breast cancer knowledge (BCWS)	0.120	0.052	0.115	0.021
CDCA score	0.019	0.048	0.020	0.686
Age ≥ 40 years	−0.534	0.279	−0.096	0.056
Nationality (Non-Kuwaiti)	0.122	0.377	—	0.747
R^2^				0.115
F (model)				6.48, *p* < 0.001

Note. CDCA = Cancer Delay and Care Avoidance—a composite measure of general help-seeking barriers adapted from the Cancer Awareness Measure (CAM) [23]; see Section 2.3.3 for full operational definition. BCWS = Breast Cancer Warning Signs knowledge score; B = unstandardized regression coefficient; SE = standard error; β = standardized regression coefficient.

## Data Availability

The data presented in this study are available on request from the corresponding author. The data are not publicly available due to privacy and ethical restrictions.

## Supplementary Materials

The following supporting information can be downloaded at: https://www.mdpi.com/article/10.3390/healthcare14121711/s1, Table S1: Item-Level Chi-Square Results: BCSBQ Item Endorsement (%) by Age Group, Education Level, and Nationality (*N* = 458).

## Abbreviations

The following abbreviations are used in this manuscript:

BCSBQ	Breast Cancer Screening Beliefs Questionnaire
BCWS	Breast Cancer Warning Signs
BSE	Breast Self-Examination
CAM	Cancer Awareness Measure
CDCA	Cancer Delay and Care Avoidance
CBE	Clinical Breast Examination
EFA	Exploratory Factor Analysis
GCC	Gulf Cooperation Council
HBM	Health Belief Model
KNMSP	Kuwait National Mammography Screening Program
ANOVA	Analysis of Variance
GLM	General Linear Model
KMO	Kaiser–Meyer–Olkin (Measure of Sampling Adequacy)
SD	Standard Deviation
SPSS	Statistical Package for the Social Sciences
