# Supplementary material for: Sociodemographic Correlates of Breast Cancer Screening Beliefs and Barriers Among Women in Kuwait: A Cross-Sectional Study Using the BCSBQ
Source: Healthcare (Basel). 2026 Jun 15;14(12):1711. doi: 10.3390/healthcare14121711 (PMC13300252; doi:10.3390/healthcare14121711)
Supplement: Supplementary file 1 [file healthcare-14-01711-s001.zip › healthcare-4250573-supplementary.pdf]

Supplementary Materials

Sociodemographic Correlates of Breast Cancer Screening Beliefs and Barriers Among Women in Kuwait: A Cross-Sectional Study Using the BCSBQ

Supplementary Table S1. Item-Level Chi-Square Results: BCSBQ Item Endorsement (%) by Age Group, Education Level, and Nationality (*N* = 458).

				Age Group			Education Level			Nationality		
Item	Subscale	Statement (original wording)	Total Endorsed (%)	<40 yrs (%)	≥40 yrs (%)	p	<Bachelor (%)	≥Bachelor (%)	p	Kuwaiti (%)	Non-Kuwaiti (%)	p
BCSB1	Positive Attitudes	I feel well, so I do not need screening	39.1%	40.3%	37.5%	0.319	38.7%	39.3%	0.354	40.4%	29.8%	0.494
BCSB2	Positive Attitudes	I follow a healthy lifestyle, so I do not need screening	29.5%	32.2%	26.0%	0.151	29.7%	29.4%	0.946	29.7%	28.1%	0.317
BCSB3	Positive Attitudes	I only go for screening when I have health problems	57.2%	57.4%	57.0%	0.911	57.4%	57.1%	0.947	56.6%	61.4%	0.079
BCSB4	Positive Attitudes	I feel healthy, so I do not need screening	44.8%	41.5%	45.6%	0.217	49.0%	42.6%	0.096	45.6%	38.6%	0.317
BCSB5	Fatalistic Beliefs	Breast cancer is a life or death sentence	10.0%	9.3%	10.5%	0.361	11.0%	9.6%	0.145	11.0%	3.5%	0.079
BCSB6	Fatalistic Beliefs	Breast cancer cannot be cured	9.2%	9.3%	9.0%	0.662	10.3%	8.6%	0.340	9.2%	8.8%	0.625
BCSB7	Fatalistic Beliefs	There is little I can do to reduce my chances of dying from breast cancer	9.2%	9.3%	9.0%	0.911	10.3%	8.6%	0.340	9.5%	7.0%	0.547
BCSB8	Fatalistic Beliefs	There is nothing I can do to change my fate	22.1%	20.9%	23.5%	0.511	20.6%	22.8%	0.603	21.7%	24.6%	0.225
BCSB9	Mammography Barriers	A mammogram will hurt my breasts	21.6%	21.7%	21.5%	0.686	25.2%	19.8%	0.187	21.7%	14.0%	0.425
BCSB10	Mammography Barriers	It is difficult to arrange transportation for a mammogram	9.6%	9.7%	9.5%	0.802	16.1%	6.3%	<0.001	9.7%	8.8%	0.524
BCSB11	Mammography Barriers	I cannot speak English	8.5%	8.9%	8.0%	0.180	16.1%	4.6%	<0.001	8.5%	8.8%	0.110
BCSB12	Mammography Barriers	I need to expose my breasts for a mammogram	20.7%	24.8%	15.5%	0.015	27.1%	17.5%	0.016	17.5%	27.1%	0.524

BCSB13	Mammography Barriers	Having a mammogram is embarrassing	27.9%	30.2%	25.0%	0.304	30.3%	26.7%	0.418	26.7%	30.3%	0.110

Notes: Endorsed (%) = proportion endorsing "agree" or "strongly agree" on the original (non-reversed) scale. Items BCSB1–BCSB4 were reverse-scored for subscale analyses; however, endorsement percentages presented here reflect original item wording. p-values reflect Pearson chi-square tests (two-sided). Bold p-values (highlighted in blue) indicate statistical significance ($p < 0.05$). Age: <40 years (n = 258) vs ≥40 years (n = 200). Education: <Bachelor's degree (n = 155) vs Bachelor's degree or higher (n = 303). Nationality: Kuwaiti (n = 401) vs Non-Kuwaiti (n = 57). Interpretation of nationality comparisons should consider the smaller non-Kuwaiti subgroup size (n = 57).
